# Relative lens vault in subjects with angle closure

**DOI:** 10.1186/1471-2415-14-93

**Published:** 2014-07-21

**Authors:** Young Kook Kim, Beong Wook Yoo, Hee Chan Kim, Tin Aung, Ki Ho Park

**Affiliations:** 1 Department of Ophthalmology, Jeju National University Hospital, Jeju, Korea; 2 Bioengineering Major, Graduate School, Seoul National University, Seoul, Korea; 3 Department of Biomedical Engineering, College of Medicine and Institute of Medical and Biological Engineering, Medical Research Center, Seoul National University, Seoul, Korea; 4 Department of Ophthalmology, Singapore National Eye Center, Singapore, Singapore; 5 Department of Ophthalmology, Seoul National University College of Medicine, Seoul, Korea; 6 Department of Ophthalmology, Seoul National University Hospital, Seoul, Korea

## Abstract

**Background:**

The purpose of this study was to investigate the association of a novel biometric parameter, relative lens vault (LV), with primary angle-closure (PAC) and primary angle-closure glaucoma (PACG).

**Methods:**

We evaluated 101 subjects with PAC (G) and 101 normal subjects that were age- and gender-matched. Based on anterior-segment optical coherence tomography scans, and using customized software, the anterior vault (AV) and LV were measured. They were defined as the maximum distances between the horizontal line connecting the two scleral spurs and the posterior corneal surface and anterior lens surface, respectively. The relative LV was calculated by dividing the LV by the AV.

**Results:**

Significant differences between PAC (G) eyes and normal eyes were found in the LV (1.06 ± 0.41 vs. 0.36 ± 0.37 mm, P < 0.001), relative LV (0.34 ± 0.23 vs. 0.11 ± 0.25, P < 0.001), and axial length (22.96 ± 0.94 vs. 24.02 ± 1.33 mm, P < 0.001). However, the two groups’ values of the AV relative to those of axial length were quite similar (both 0.14 ± 0.03, *P* = 0.91). The relative LV values distinguished between PAC (G) eyes and normal eyes better than the LV values (area under the receiver operator characteristic curve: 0.97 vs. 0.92, P = 0.032).

**Conclusions:**

Our results suggest that relative dimensions of the eyeball’s anterior portion in PAC (G) eyes might be within the normal range. And the value of LV relative to that of the AV (i.e., the relative LV) is more closely related to PAC (G) than is the absolute value of LV.

## Background

The scleral spur, marked by a prominent inner extension of the sclera on anterior-segment optical coherence tomography (AS-OCT) is an important anatomical landmark in quantitative anterior chamber angle measurements [1]. It serves as a reference point determining the relative position of the trabecular meshwork and of parameters such as the angle-opening distance, the angle-recess area, and the trabecular-iris area [2,3]. Recently, the lens vault (LV), also determined relative to the scleral spur, was introduced as a novel biometric-parameter [4]. The LV, defined as the perpendicular distance between the horizontal line joining the two scleral spurs and the anterior pole of the crystalline lens [4], represents the anterior portion of the lens, and, as such, is strongly and independently associated with angle closure [5]. An increased LV indicates an increased lens thickness and/or bulk anterior to the scleral spur plane, which subsequently increases the risk of angle closure.

Many studies on different populations have shown the LV to be associated with angle-closure risk [4-8]. However, the possibility of the LV affecting angle closure differently according to the anterior chamber depth (ACD) is overlooked. If the ACD is deep enough, the LV, even when large, would be less likely to increase the risk of angle-closure. Conversely, if the patient’s anterior chamber is shallow, the angle-closure risk would relatively increase even when the LV is small. These hypotheses suggest that LV relative values (to the anterior chamber) may be more closely related to angle-closure risk than are absolute values.

In the present study, we investigated, based on AS-OCT scans, two novel biometric parameters: the anterior vault (AV), which represents the sum of the LV and the ACD, and the relative LV (rLV), which represents the ratio of the LV to the AV. Additionally, we aimed to evaluate the importance of the rLV in distinguishing between eyes with and without angle closure.

## Methods

All of the study participants were examined between January 2010 and August 2013 at the glaucoma and cataract service of Seoul National University Hospital, Seoul, Korea. All eligible participants were consecutively enrolled by retrospective medical-record review. At the initial clinic visit, all underwent a complete ophthalmic examination, including medical history review, best-corrected visual acuity measurement, slit-lamp biomicroscopy, Goldmann applanation tonometry (Haag-Streit, Koniz, Switzerland), funduscopic examination (90 diopter lens), stereoscopic optic disc photography, retinal nerve fiber layer photography, and gonioscopy, performed in the dark using a Sussman 4-mirror lens at high magnification (x16). Indentation gonioscopy with the same lens was used to establish the presence or absence of peripheral anterior synechiae. The cataract type and grade was evaluated, based on the Lens Opacity Classification System II [9]. Additionally, AS-OCT imaging (Visante, Carl Zeiss Meditec, Dublin, CA) and axial length (AL) measurement (IOL Master; Carl Zeiss Meditec) were performed at the initial or follow-up clinic visit.

This study, approved by the Seoul National University Hospital institutional review board adhered to the tenets of the Declaration of Helsinki. Informed consent was obtained from all subjects.

### Angle-closure group and normal-control group classification

Angle closure was defined as primary angle-closure (PAC) or primary angle-closure glaucoma (PACG). The PAC, PACG, and open-angle criteria used were as follows:

•PAC: the pigmented posterior trabecular meshwork was not visible on nonindentation gonioscopy for 180 degrees or more in the primary position, with peripheral anterior synechiae and/or raised intraocular pressure (IOP), and no glaucomatous optic neuropathy [10].

•PACG: PAC associated with glaucomatous optic neuropathy (defined as loss of neuroretinal rim with vertical cup-to-disc ratio of >0.7 or inter-eye asymmetry of >0.2, and/or presence of glaucoma-associated notching) [10].

•Open angle: the pigmented posterior trabecular meshwork was visible on nonindentation gonioscopy for 180 degrees or more in the primary position, with the IOP ≤21 mmHg, and no peripheral anterior synechiae/glaucomatous optic neuropathy [10].

Patients were excluded if they had undergone prior intraocular surgery or if their AS-OCT images were of a quality sufficiently poor to make scleral spur or anterior lens surface identification difficult. All of the subjects with PAC(G) previously had undergone laser peripheral iridotomy.

Subjects who had PAC(G) and met the relevant eligibility criteria made up the PAC(G) group; subjects who had open angles and met those eligibility criteria made up the normal-control group, and were randomly age- and gender-matched, one-by-one, to the PAC(G) subjects. In cases where both eyes met all of the eligibility criteria, one eye was randomly selected as the study eye.

### Anterior-segment optical coherence tomography

All of the subjects underwent AS-OCT imaging by a single operator in a dark room without any illuminating device (except for the monitor). The scans were centered on the pupil; one cross-sectional horizontal scan (nasal-temporal angles: 0°–180°) was evaluated for each subject. In order to obtain the best-quality image, the operator adjusted the image saturation and noise and optimized the polarization prior to initiating the scan collection.

### Anterior-segment optical coherence tomography parameters

**Figure 1:**
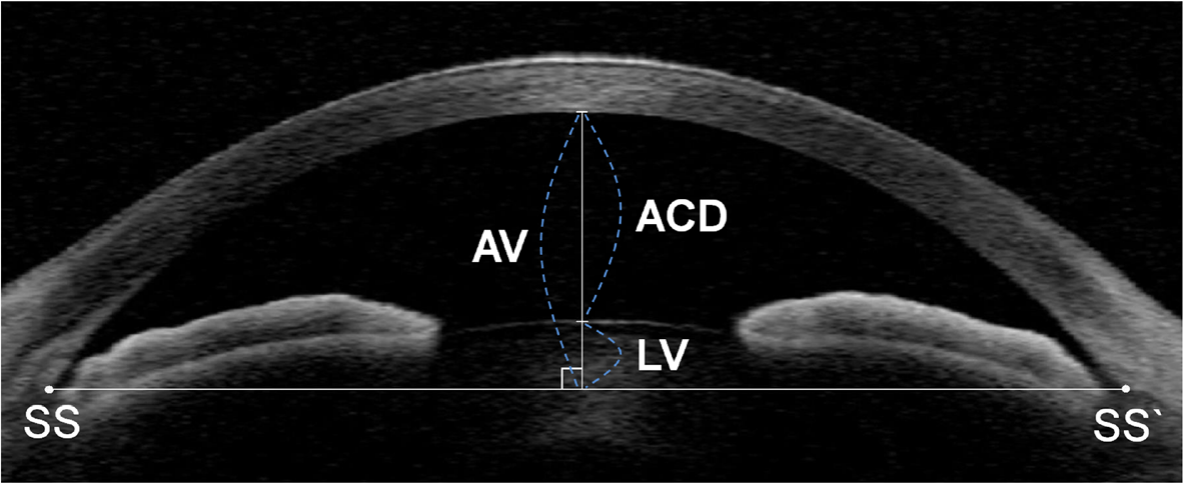
**Anterior-segment optical coherence tomography (AS-OCT) image illustrating measurement of anterior chamber depth (ACD), lens vault (LV), and anterior vault (AV) by Seoul Anterior-Segment Assessment Program (SAAP).** Points SS and SS’ indicate the sclera spur positions. The relative LV (rLV) was calculated by dividing the LV by the AV.

The AS-OCT measurement parameters, summarized in Figure 1, were defined as follows:

•The AV: the maximum distance, measured perpendicular to the horizontal line connecting the two scleral spurs, between that line and the posterior corneal surface on horizontal AS-OCT scans.

•The LV: the maximum distance, measured perpendicular to the horizontal line connecting the two scleral spurs, between that line and the anterior lens surface on horizontal AS-OCT scans.

•The ACD: the maximum distance, measured perpendicular to the horizontal line connecting the two scleral spurs, between the posterior corneal surface and the anterior lens surface, on horizontal AS-OCT scans.

•Estimated AV: the value obtained by summing the LV and ACD values.

•The rAV: the AV divided by the AL.

•The rLV: the LV divided by the AV.

### Image processing and Seoul anterior-segment assessment program

The AS-OCT scans were processed using the Seoul Anterior-Segment Assessment Program (SAAP, Medical Electronics Lab of Seoul National University, Seoul, Korea), a customized software in Matlab (2013a version, The MathWorks, Inc., Natick, MA, USA). They were independently performed by two ophthalmologists (Y.K.K., K.H.P.) masked to clinical data.

The SAAP automatically extracted 1200 × 600 32-bit color scale image portions of the output file and performed noise and contrast conditioning. A 3 mm-sized scale bar marked on the AS-OCT scan image was detected automatically for calibration purposes. Then, the color scale image was converted to grey scale (intensities: 0 ~ 255). A binary copy of the image was produced, with pixels defined as 1 s (tissue) or 0 s (open space) according to a calculated brightness/darkness threshold value. The two observers independently determined the locations of the two scleral spurs, which appear as an inward sclera protrusion, as well as the two points on the anterior lens surfaces, which are the nearest points to the pupillary margin. Then, a hole-filling algorithm defined the borders of the corneal endothelium and the anterior surface of the lens. Further, three anterior-segment parameters (AV, LV, and ACD) were automatically calculated.

The SAAP automatically extracted 1200 × 600 32-bit color scale image portions of the output file and performed noise and contrast conditioning. A 3 mm-sized scale bar marked on the AS-OCT scan image was detected automatically for calibration purposes. Then, the color scale image was converted to grey scale (intensities: 0 ~ 255). A binary copy of the image was produced, with pixels defined as 1 s (tissue) or 0 s (open space) according to a calculated brightness/darkness threshold value. The two observers independently determined the locations of the two scleral spurs, which appear as an inward sclera protrusion, as well as the two points on the anterior lens surfaces, which are the nearest points to the pupillary margin. Then, a hole-filling algorithm defined the borders of the corneal endothelium and the anterior surface of the lens. Further, three anterior-segment parameters (AV, LV, and ACD) were automatically calculated.

To determine the reproducibility of the SAAP measurement of the three AS-OCT parameters, the intraclass correlation coefficient (ICC) To determine the reproducibility of the SAAP measurement of the three AS-OCT parameters, the intraclass correlation coefficient (ICC) [11] and the coefficient of variation (COV) were analyzed for the PAC(G) and normal-control groups. The numbers of participants were calculated for the 95% lower confidence interval (CI) of ICC 0.8 so as not to be lower than 0.75, a generally accepted lower value for good reproducibility [12]. The COV was defined as the standard deviation (SD) divided by the average of each of the AS-OCT parameters of each set, expressed as a percentage. Two independent observers (both glaucoma fellowship-trained ophthalmologists), referencing a total of 90 AS-OCT images (45 PAC[G] images, 45 normal-control images), measured the AS-OCT parameters three times. The ICC for the three AS-OCT parameters was between 0.975 and 0.988 in the intraobserver evaluation, and between 0.962 and 0.981 in the interobserver evaluation; the COV was between 1.9% and 3.3% in the intraobserver evaluation and between 2.0% and 4.0% in the interobserver evaluation (Table 1). The SAAP showed excellent intraobserver and interobserver reproducibility of the AS-OCT measurement parameters (lowest ICC: 0.962; highest COV: 4.0%).

**Table 1:** Intraclass correlation coefficient and coefficient of variation of intraobserver and interobserver evaluations

	**Intraobserver**		**Interobserver**	
	**ICC**	**COV (%)**	**ICC**	**COV (%)**
LV	0.988 (0.983)	1.9	0.980 (0.972)	2.0
ACD	0.975 (0.965)	3.3	0.981 (0.973)	3.4
Anterior vault	0.977 (0.968)	2.9	0.962 (0.945)	4.0

ICC = Intraclass Correlation Coefficient with Lower 95% confidence interval; COV = Coefficient of Variation; LV = lens vault; ACD = anterior chamber depth.

### Statistical analysis

Statistical analyses were performed using SPSS software (version 19, SPSS, Inc., Chicago, IL) and MedCalc software (version 12.7.5.0, Mariakerke, Inc., Belgium). The differences between the two groups in the mean values of the parametric data were examined using the independent samples Student t-test. Receiver operator characteristic curves were generated, and the area under the receiver operator characteristic curve (AUROC) was used to assess the performance of the various parameters in detecting angle closure. The sensitivity and specificity were calculated, using the optimal cutoff point, based on the maximum of the Youden index (calculated as J = max [sensitivity + specificity – 1]) [13]. A *P* value < 0.05 was considered to be statistically significant.

## Results

A total of 1,335 Korean subjects (124 PAC[G] patients and 1,196 control subjects) who fulfilled the required examination data were recruited for the study. Among the 124 PAC(G) patients, 64 (51.6%) were found to have peripheral anterior synechiae. Seven of the 64 were excluded due to low-quality AS-OCT images on which the scleral spur could not be clearly defined, and 5 subjects were excluded due to an unclear anterior lens surface. The qualifying 112 PAC(G) subjects were each matched randomly to one of the 1,196 control candidates of the same age and gender. Of the 112 control subjects matched, 11 had low quality of AS-OCT image to clearly define anterior lens surface or scleral spur, and so were excluded. Consequently, 101 PAC(G) subjects and 101 age- and gender-matched control subjects were the final cohorts included in the study. In both groups, the average patient age was 64.5 ± 6.2 years, and 46 of 101 subjects (45.6%) were male. And there was no significant difference between the groups either in cataract type or grade distribution (*P* = 0.83). Likewise, there was no significant difference in the best-corrected visual acuity or spherical equivalent (*P* = 0.79 and 0.66, respectively).

Compared with the normal eyes, the PAC(G) eyes had significantly larger LV values (1.06 ± 0.41 vs. 0.36 ± 0.37 mm, *P* < 0.001), shallower anterior chambers (ACD = 2.06 ± 0.40 vs. 2.94 ± 0.38 mm, *P* < 0.001), and shorter AL (22.96 ± 0.94 vs. 24.02 ± 1.33 mm, *P* < 0.001) (Table 2). Examination of the novel parameters revealed that the PAC(G) eyes had significantly larger rLV values (0.34 ± 0.23 vs. 0.11 ± 0.25, *P* < 0.001). The AV values were smaller in the PAC(G) eyes but without significance (3.11 ± 0.77 vs. 3.28 ± 0.79 mm, *P* = 0.12). Interestingly, the two groups’ rAV values were quite similar (both 0.14 ± 0.03, *P* = 0.91).

**Table 2:** Known and novel parameters of study subjects

		**PAC (G) (n = 101) mean (SD)**	**Normal control (n = 101) mean (SD)**	***P *** **value**
Known parameters	LV (mm)	1.06 (0.41)	0.36 (0.37)	<0.001
	ACD (mm)	2.06 (0.40)	2.94 (0.38)	<0.001
	Axial length (mm)	22.96 (0.94)	24.02 (1.33)	<0.001
Novel parameters	AV (mm)	3.11 (0.77)	3.28 (0.79)	0.12
	Estimated AV^*^ (mm)	3.12 (0.80)	3.30 (0.76)	0.10
	Relative LV^**^	0.34 (0.23)	0.11 (0.25)	<0.001
	Relative AV^***^	0.14 (0.03)	0.14 (0.03)	0.907

PAC (G) = primary angle-closure (glaucoma); SD = standard deviation; LV = lens vault; ACD = anterior chamber depth; AV = anterior vault.

^*^Estimated anterior vault = LV + ACD.

^**^Relative LV = LV/AV.

^***^Relative anterior vault = AV/Axial length.

**Figure 2:**
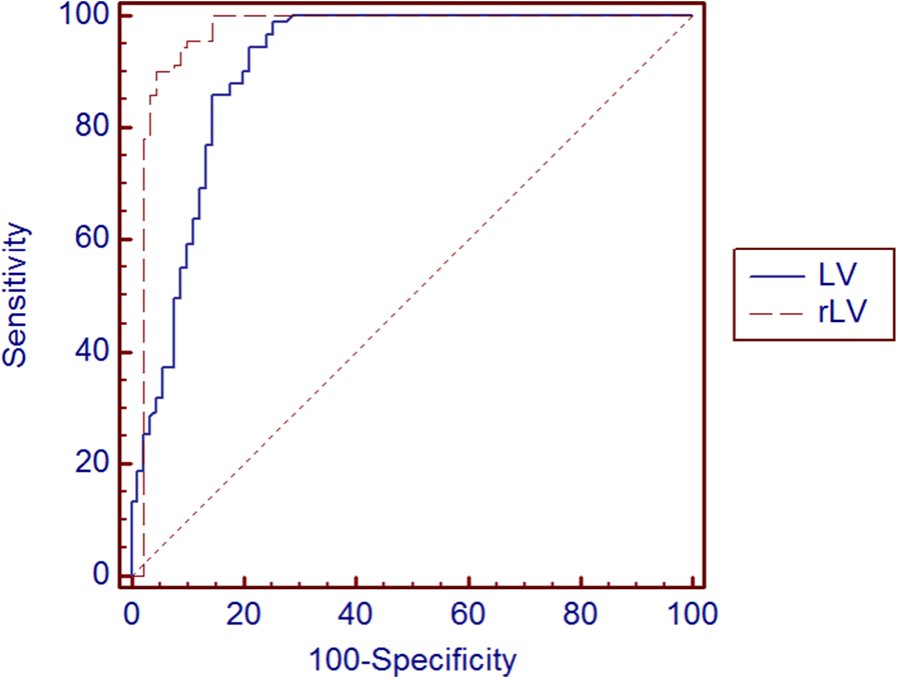
**Areas under the receiver-operating characteristic curves (AUROCs) of lens vault (LV) and relative LV (rLV) for distinguishing primary angle-closure (PAC) or primary angle-closure glaucoma PACG) subjects from normal subjects.** The AUROCs significantly differed between the rLV and the LV (*P* = 0.032).

In terms of distinguishing PAC(G) subjects from normal subjects, the rLV showed the highest AUROC (0.97, 95% CI = 0.93–0.99), a sensitivity of 98.7%, and a specificity of 86.0%. The AUROC of the LV was 0.92, and the other lens-parameter AUROCs ranged from 0.73 to 0.83 (Table 3). The AUROCs significantly differed between the rLV and LV (*P* = 0.032) as well (Figure 2).

**Table 3:** Area under the receiver operating characteristic curve, sensitivity and specificity for known and novel angle-closure-detection parameters for detecting angle closure

		**AUROC (95% CI)**	**Optimal Cutoff Point**^**+**^	**Sensitivity**^**++ **^**(95% CI)**	**Specificity**^**++ **^**(95% CI)**
Known Parameters	LV	0.92 (0.86, 0.96)	0.75	95.2 (91.1, 98.5)	74.6 (64.5, 84.0)
	ACD	0.83 (0.76, 0.88)	0.63	88.3 (85.7, 92.1)	73.7 (66.2, 78.0)
	Axial length	0.73 (0.67, 0.80)	0.53	72.0 (67.1, 77.5)	68.8 (62.4, 74.3)
Novel Parameters	Anterior vault	0.76 (0.67, 0.81)	0.60	83.7 (74.2, 86.7)	71.1 (67.7, 74.6)
	Relative LV^*^	0.97 (0.93, 0.99)	0.85	98.7 (94.3, 100.0)	86.0 (76.9, 91.5)

AUROC = area under the receiver operating characteristic curve; CI = confidence interval; LV = lens vault; ACD = anterior chamber depth.

^*^Relative LV = LV/Anterior vault.

^+^given by the maximum of the Youden index, calculated as J = max(sensitivity + specificity-1).

^++^sensitivity and s.

## Discussion

**Figure 3:**
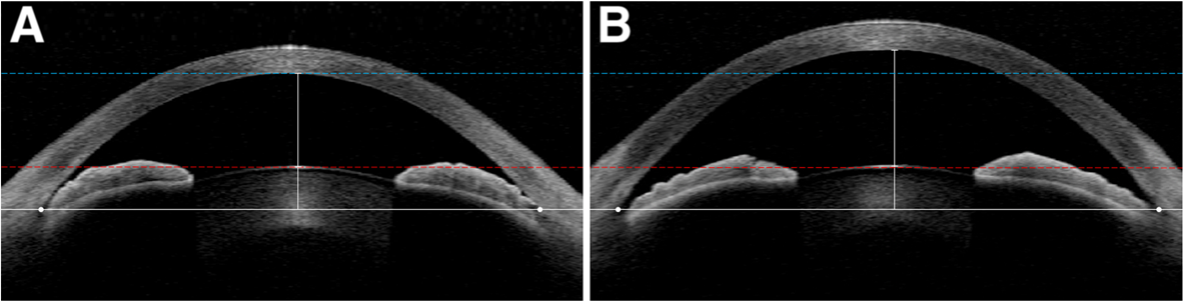
**Examples of (A) PAC (G) and (B) normal-control eyes with similar lens vault (LV).** The white horizontal lines connect the two scleral spurs (white points). **(A)** The LV is 1.03 mm, the anterior chamber depth (ACD) is 2.51 mm, and the anterior vault (AV) is 3.53 mm, in this eye with PAC (G). **(B)** The LV is 1.04 mm, the ACD is 3.19 mm, and the AV is 4.23 mm, in this eye with open-angle. The LV values are similar; however, due to the ACD difference, the relative LV values differ between **(A)** and **(B)** (0.29 in PAC[G] vs. 0.25 in normal-control eye).

Consistently with previous studies [4,7], the LV, in the present study, is significantly larger in PAC(G) eyes than in normal eyes. Additionally, the rLV, which we suggest herein as a novel parameter, also was significantly larger in the PAC(G) eyes than in the normal eyes (*P* < 0.001), and was better for detecting PAC(G) than the LV (rLV AUROC = 0.97, LV AUROC = 0.92, *P* = 0.032). This supports the idea that the LV value relative to that of the AV (i.e., the rLV) is more closely related to angle-closure risk than the absolute value of LV (Figure 3).

Recent studies suggest that in the pathogenesis of angle closure, multiple anatomical and physiological factors interact. For instance, it was found that the iris is more convex, has a larger area, and is thicker in eyes with narrow angles, which resulted in more-anterior bowing and crowding of the peripheral iris [14]. Other anatomic parameters, such as smaller anterior chamber width, area, and volume, are independently associated with increased risk of angle closure [4,15]. The fact that iris volume dynamically increases during pupil dilation supports the theory that some patients show physiological predisposition to angle closure [16,17]. Choroidal expansion has been observed in both untreated and treated acute and chronic primary-angle closure [18]. Of the multiple factors associated with PAC(G) pathogenesis, the LV is determined by lens thickness and position, but the rLV is influenced also by the AL and ACD. Therefore, the rLV reflects the combined effects of several more factors than does the LV.

The rLV and AV parameters are of potential clinical value. First, with the building of normative rLV and AV databases, it could be used as a screening tool in predicting a patient’s PAC risk. Moreover, by knowing each patient’s AV, it might be possible to predict the extent to which the ACD decreases as the LV increases.

In comparing the estimated-AV with the AV in the present study, the former was slightly larger (3.12 ± 0.80 vs. 3.11 ± 0.77 in PAC[G]; 3.30 ± 0.76 vs. 3.28 ± 0.79 in normal control), though the differences were not statistically significant (*P* = 0.89 and 0.77, respectively). Because the ACD is the distance from the anterior lens pole to the posterior corneal surface, and the LV is the distance from the anterior lens pole to the horizontal line connecting the two scleral spurs, the sum of the ACD and LV should, theoretically, be equal to the AV. Unfortunately however, the anterior pole of the lens, the posterior pole of the corneal surface, and the perpendicular line from the horizontal line connecting the two scleral spurs do not always line up. Therefore, small discrepancies between estimated AV and measured AV are to be expected, and were observed in this study.

The rLV and AV parameters are of potential clinical value. First, with the building of normative rLV and AV databases, it could be used as a screening tool in predicting a patient’s PAC risk. Moreover, by knowing each patient’s AV, it might be possible to predict the extent to which the ACD decreases as the LV increases.

In comparing the estimated-AV with the AV in the present study, the former was slightly larger (3.12 ± 0.80 vs. 3.11 ± 0.77 in PAC[G]; 3.30 ± 0.76 vs. 3.28 ± 0.79 in normal control), though the differences were not statistically significant (*P* = 0.89 and 0.77, respectively). Because the ACD is the distance from the anterior lens pole to the posterior corneal surface, and the LV is the distance from the anterior lens pole to the horizontal line connecting the two scleral spurs, the sum of the ACD and LV should, theoretically, be equal to the AV. Unfortunately however, the anterior pole of the lens, the posterior pole of the corneal surface, and the perpendicular line from the horizontal line connecting the two scleral spurs do not always line up. Therefore, small discrepancies between estimated AV and measured AV are to be expected, and were observed in this study.

An interesting finding was that the rAV was quite similar between the PAC(G) eyes (0.14 ± 0.03) and the normal eyes (0.14 ± 0.03 mm, *P* = 0.91). Based on the results of previous studies [6,7], we attempted to compute the estimated rAV (the value obtained by dividing the estimated AV by the AL), as shown in Table 4. As with our data, the values of the estimated rAV were very coterminous between the angle-closure eyes and normal eyes. That is, although the AL in the PAC(G) group was significantly smaller than in the control group, the ratio of the AV to the AL did not much differ. These results suggest that the relative dimensions of the eyeball’s anterior portion in PAC(G) eyes might be within the normal range.

**Table 4:** Estimated anterior vault (AV) and relative AV from previous studies

	**Angle closure vs. Normal control**					
	**Moghimi et al. **[16]** [Iranian]**		**Moghimi et al. **[16]** [Iranian]**		**Ozaki et al. **[7]** [Japanese]**	
	**AACG**	**Fellow eye**	**PACS**	**Control**	**AC**	**NC**
LV (mm)	1.07 (0.25)	0.98 (0.19)	0.89 (0.22)	0.27 (0.31)	1.0 (0.26)	0.49 (0.24)
ACD (mm)	2.26 (0.22)	2.36 (0.21)	2.53 (0.28)	3.15 (0.33)	2.51 (0.39)	3.14 (0.35)
Axial length (mm)	21.84 (1.17)	21.69 (1.13)	21.97 (0.73)	22.46 (4.35)	22.22 (0.77)	23.28 (0.81)
Estimated AV^*^ (mm)	3.33	3.34	3.42	3.42	3.51	3.63
Estimated relative AV^**^	0.15	0.15	0.16	0.15	0.16	0.15

AACG = acute angle-closure glaucoma; PACS = primary angle-closure suspect; AC = angle closure; NC = normal control; LPI = laser peripheral iridotomy; LV = lens vault; ACD = anterior chamber depth; AV = anterior vault.

ACD, LV, and Anterior vault are given as mean values ± standard deviation.

^*^Estimated AV = LV + ACD.

^**^Estimated relative AV = Estimated AV/Axial length.

This study can be distinguished from the above-noted earlier one [4] in several respects. First, our control group was, patient-by-patient, both age- and gender-matched; as such, there were no cataract-type or grade-distribution differences between diseased eyes and normal-control eyes. Thus the possible influences of age, gender, and cataract on the LV and ACD values were minimized. Second, whereas in previous studies, the LV (by AS-OCT) and the ACD (by A-scan biometry) were measured with different imaging modalities, in our study, both the LV and the ACD were measured on a single AS-OCT with customized software (SAAP). Thereby, measurement errors were reduced.

It should be noted that the present study also has some limitations. First, the patients were recruited from a single tertiary referral hospital. Second, the imaging measurements proceeded retrospectively. Third, the AS-OCT images were collected following laser peripheral iridotomy on all PAC(G) eyes. An AS-OCT-imaging study conducted prior to laser peripheral iridotomy might be more meaningful for assessment of PAC(G) risk factors, because it is possible that laser peripheral iridotomy influences the anterior chamber structure. Further, it is not possible to compare the diagnosis ability of the rLV to those of parameters such as the angle opening distance, trabecular iris area, and angle recess area. The last limitation is that we did not separate the PAC and PACG subjects in study design. In future, further, longitudinal-design studies are needed in order to observe how the rLV and AV change during the PAC(G) disease process or over the course of aging.

## Conclusions

We found that eyes with PAC(G) have, relative to normal eyes, larger rLV, a parameter distinguished the two groups better than LV. These findings suggest that, because the LV can affect PAC(G) development differently according to the ACD, the value of LV relative to that of the AV (i.e., the rLV) is more closely related to PAC(G) than is the absolute value of LV. The two groups’ rAV values, which represent the ratio of the AV to the AL, were quite similar. That is, the relative position of the scleral spur throughout the antero-posterior dimension of the eyeball seems to be constant regardless of whether eyes are PAC(G) or normal.

## Abbreviations

AV: Is used for ‘anterior vault’; LV: Is used for ‘lens vault’; PAC: Is used for ‘primary angle-closure’; PACG: Is used for ‘primary angle-closure glaucoma’; AS-OCT: Is used for ‘anterior-segment optical coherence tomography’; ACD: Is used to abbreviate ‘anterior chamber depth’; AL: Is used to abbreviate ‘axial length’; IOP: Is used to abbreviate ‘intraocular pressure’; SAAP: Is used to abbreviate ‘Seoul Anterior-Segment Assessment Program’; ICC: Is used to abbreviate ‘intraclass correlation coefficient’; COV: Is used to abbreviate ‘coefficient of variation’; CI: Is used to abbreviate ‘confidence interval’; SD: Is used to abbreviate ‘standard deviation’; AUROC: Is used to abbreviate ‘area under the receiver operator characteristic curve’.

## Competing interests

The authors declare that they have no competing interests.

## Authors’ contributions

YKK contributed to study design and coordination, conducted the data analysis, and assisted in drafting and revising the manuscript. BWY, HCK, and TW were involved in data acquisition, data interpretation, and critical revision of the manuscript. KHP helped to interpret the data and to draft and revise the manuscript. All authors have given final approval of the version to be published.

## Pre-publication history

The pre-publication history for this paper can be accessed here:

http://www.biomedcentral.com/1471-2415/14/93/prepub
